# Comparison of neuroprotective efficacy of poly-arginine R18 and R18D (D-enantiomer) peptides following permanent middle cerebral artery occlusion in the Wistar rat and *in vitro* toxicity studies

**DOI:** 10.1371/journal.pone.0193884

**Published:** 2018-03-07

**Authors:** Diego Milani, Megan C. Bakeberg, Jane L. Cross, Vince W. Clark, Ryan S. Anderton, David J. Blacker, Neville W. Knuckey, Bruno P. Meloni

**Affiliations:** 1 Perron Institute for Neurological and Translational Sciences, Nedlands, Australia, Western Australia, Nedlands, Western Australia, Australia; 2 Department of Neurosurgery, Sir Charles Gairdner Hospital, Nedlands, Western Australia, Australia; 3 School of Heath Sciences and Institute for Health Research, The University Notre Dame Australia, Fremantle, Western Australia, Australia; 4 Centre for Neuromuscular and Neurological Disorders, The University of Western Australia, Nedlands, Australia; 5 Department of Neurology, Sir Charles Gairdner Hospital, Nedlands, Western Australia, Australia; Albany Medical College, UNITED STATES

## Abstract

We have previously demonstrated that arginine-rich and poly-arginine peptides possess potent neuroprotective properties, with poly-arginine peptide R18 identified as being highly effective at reducing infarct volume following middle cerebral artery occlusion (MCAO) in the Sprague Dawley rat. Since peptides synthesised using D-isoform amino acids have greater stability than L-isoform peptides due to increased resistance to proteolytic degradation, they represent potentially more effective peptide therapeutics. Therefore we compared the neuroprotective efficacy of R18 and its D-enantiomer R18D following permanent MCAO in the Wistar rat. Furthermore, as increased peptide stability may also increase peptide toxicity, we examined the effects of R18 and R18D on cultured cortical neurons, astrocytes, brain endothelial cells (bEND.3), and embryonic kidney cells (HEK293) following a 10-minute or 24-hour peptide exposure duration. The *in vivo* studies demonstrated that R18D resulted in a greater reduction in mean infarct volume compared to R18 (33%, *p =* 0.004 vs 12%, *p =* 0.27) after intravenous administration at 300 nmol/kg 30 minutes after MCAO. Both R18D and R18 reduced cerebral hemisphere swelling to a comparable degree (27%, *p =* 0.03 and 30%, *p =* 0.02), and improved neurological assessment scores (1.5, *p =* 0.02 and 2, *p =* 0.058 vs 3 for vehicle). No abnormal histological findings specific to peptide treatments were observed in hematoxylin and eosin stained sections of kidney, liver, spleen, lung and heart. *In vitro* studies demonstrated that R18 and R18D were most toxic to neurons, followed by astrocytes, HEK293 and bEND.3 cells, but only at high concentrations and/or following 24-hour exposure. These findings further highlight the neuroprotective properties of poly-arginine peptides, and indicate that R18D at the dose examined is more potent than R18 in Wistar rats, and justify continued investigation of the R18 peptide as a novel neuroprotective agent for stroke.

## Introduction

Stroke is a major cause of death and disability globally, with no clinically effective pharmacological neuroprotective treatments available to reduce brain injury and improve patient outcomes. We have previously demonstrated that cationic arginine-rich and poly-arginine peptides (here after referred to as CARPs) possess potent neuroprotective properties in both *in vitro* and *in vivo* stroke related injury models [1–10] with peptide arginine content and peptide positive charge being critical for neuroprotection [2,3]; findings confirmed by others [11,12]. Furthermore, as an important feature of any central nervous system therapeutic, CARPs on their own or when fused to cargo molecules (e.g. other peptides, proteins, nucleic acids and drugs) have the capacity to cross the blood brain barrier (BBB) and enter into the brain and neural cells [13–17].

Based on *in vitro* studies [2] we have identified poly-arginine peptides R12, R15 and R18 (12-, 15- and 18-mer of arginine respectively; R = arginine) as being particularly neuroprotective, and following a comparative study [5] of the three peptides using a permanent middle cerebral artery occlusion (MCAO) model, selected R18 (net charge +18) as our lead peptide. Furthermore, additional studies [6,7] using both transient and permanent middle cerebral artery stroke models demonstrated that R18 has superior neuroprotective efficacy than the previously characterised NR2B9c peptide fused to the cationic cell penetrating peptide TAT (TAT-NR2B9c; now known as NA-1) [18,19]. The NA-1 peptide is a CARP (YGRKKRRQRRR-KLSSIESDV; net charge +7 charge) and is currently being assessed in two Phase 3 clinical stroke trials [20,21]. Therefore, while R18 already represents a potent neuroprotective agent, if its neuroprotective potency was enhanced even further it could improve its clinical effectiveness for stroke, and potentially for other acute and chronic neurodegenerative disorders. Given that peptides synthesised with D-isoform amino acids have an increased resistance to proteolytic degradation and consequent higher serum stability [22–24] they represent potentially even more effective therapeutic agents.

We have previously confirmed that D-isoform poly-arginine peptides are neuroprotective in both *in vitro* and *in vivo* stroke injury models. For example, we have demonstrated that poly-arginine peptide D-enantiomers R9D and R18D are highly neuroprotective following *in vitro* glutamic acid excitotoxicity [2,9,10], and also reduce infarct volume after permanent MCAO and hypoxia-ischemic encephalopathy (HIE) in the Sprague Dawley rat [2,10], respectively. Comparative studies of R18 and R18D in the excitotoxicity and HIE models showed that the two isomers had a similar degree of neuroprotective efficacy and capacity to inhibit neuronal intracellular calcium influx [10].

With respect to neuroprotective mechanisms of action we have previously shown that CARP’s including both R18 and R18D, have the capacity to reduce glutamic acid induced excitotoxic neuronal death and intracellular calcium influx [2–4,9,10]. In addition, we have demonstrated that the poly-arginine peptide R12 and the NA-1 peptide reduce neuronal surface expression of the NMDA receptor subunit protein NR2B9c [8]. The ability of CARPs to reduce cell surface levels of NR2B is at least one mechanism whereby the peptides reduce the damaging effects of glutamate-induced excitotoxicity. Furthermore, based on other CARP neuroprotection studies, which include many TAT-fused putative neuroprotective peptides, they have been demonstrated to down-regulate and/or antagonise other neuronal calcium channels/receptors [12, 25–34], activate pro-survival signalling pathways in neurons [34–36], assist in maintaining neuronal mitochondrial integrity [11,37] and reduce neuro-inflammation [38,39]. In addition, it is likely that the ability of CARPs to exert their neuroprotective actions is mediated in part, to the positively charged guanidino group that is only present in arginine residues [2,3,11,12]. The gaunidino head group can form electrostatic interactions with positively charged structures on plasma and mitochondrial membranes (e.g. phospholipid head groups, heparin sulphate proteoglycans, receptors, sialic acid residues) as well as, possessing anti-oxidant properties [40–43]. Given that CARPs have several potential neuroprotective mechanisms of action is highly significant, as it is widely considered that to obtain neuroprotective efficacy at the clinical level will require a combination of treatments or a treatment targeting two or more neurodamaging and/or neuroprotective pathways.

Considering the potential application for R18 and R18D as future neuroprotective therapeutics for stroke, the present study had several objectives. The first was to compare the effectiveness of R18 and R18D in a permanent MCAO stroke model. Furthermore, as MCAO stroke severity and neuroprotective treatment effects may differ in different strains of rats [44–46] and given that previous studies in our laboratory have only assessed CARPs in Sprague Dawley rats, the present study used Wistar rats. In addition, to obtain a measure of potential cellular toxicity, the adverse effects of R18 and R18D were examined in peripheral tissues (i.e. kidney, liver, spleen, lung and heart) collected from rats treated with the peptides, and following exposure of the peptides to cultured cells (i.e. cortical neurons, astrocytes, brain endothelial cells and embryonic kidney cells).

## Material and methods

### Peptides used in the study

The R18 (H-RRRRRRRRRRRRRRRRRR-OH) and R18D (H-rrrrrrrrrrrrrrrrrr-OH) peptides used in the animal study were synthesised containing the L-isoform and the D-isoform amino acids (D-arginine represented by lower case) respectively. Peptides were synthesised by Mimotopes (Australia), purified by high performance liquid chromatography to at least 98% purity, and subjected to peptide hydrolysis and amino acid liquid chromatography analysis to obtain a precise measure of peptide content (Mimotopes). For *in vitro* studies peptides were prepared in water for irrigation (Baxter, Australia) as a 500 μM stock, and for animal studies in 0.9% sodium chloride for injection (Pfizer, Perth, Australia) in a 650 μl volume in 3 mL syringes. Reconstituted peptides were stored at -20°C until use.

### Permanent middle cerebral artery occlusion surgical procedure

This study was approved by the Animal Ethics Committee of the University of Western Australia (Approval number: RA/3/100/1362) and follows the guidelines outlined by the *Australian Code for the Care and Use of Animals for Scientific Purposes*. The surgical procedure for performing the permanent middle cerebral artery occlusion (MCAO) stroke model in rats, induced using an intraluminal monofilament, has been described previously [5,6]. Briefly, healthy male Wistar rats weighing 280–320 g were housed under controlled conditions; 12 hour light/dark cycle, free access to food (rat chow) and water and environmental enrichment (tissue/paper towels, gnawing blocks and red plastic cylinder in cage). Rats were purchased from the Animal Resources Centre, Murdoch, Western Australia.

Experimental animals, that had been fasted overnight, were anesthetised with the use of a facemask with 4% isoflurane (mix 30% oxygen/70% nitrous oxide) and anesthetic maintenance with 2–3% isoflurane. A laser Doppler probe was attached 1 mm posterior to the bregma and 4 mm lateral from the midline of the skull to measure cerebral blood flow. The tail artery was cannulated to allow blood pressure monitoring and withdrawal of blood samples for measurement of arterial blood gases (PaO_2_, PaCO_2_), pH (ABL5, Radiometer, Copenhagen, Denmark) and plasma glucose levels (MediSense Optium, Abbott Laboratories, USA). The MCAO procedure was considered successful if there was at least 25% decrease from baseline in cerebral blood flow (CBF) after insertion of the filament to occlude the right middle cerebral artery, as measured by laser Doppler flowmetry. In addition, using a rectal probe (Physitemp Instruments, USA), the animal body temperature was closely monitored during surgery and maintained between 37–37.8°C, with fan heating or cooling, as required. Treatments were administered intravenously through the right internal jugular vein, thirty minutes after MCAO, using an infusion pump (600 μl over 6 minutes).

Treatment groups consisted of R18 and R18D at 300 nmol/kg, and vehicle (0.9% sodium chloride). The 300 nmol/kg dose was selected because in previous studies examining the effectiveness of R18 in permanent and transient MCAO stroke, this dose reduced infarct volume to a lesser extent than a 1000 nmol/kg dose, thus if R18D was more neuroprotective than R18, the 300 nmol/kg dose would increase the likelihood of being able to demonstrate a difference in the efficacy of the L- and D-isoforms. Treatments were randomised and all procedures were performed while being blinded to treatments.

### Post-surgical analgesia and animal monitoring

At the conclusion of surgery, pethidine was administered intramuscularly (1 mg in 0.2 mL saline) and bupivacaine was administered subcutaneously (0.1 mg in 0.2 mL saline per site) to tail and head surgical wounds. After surgery, the body temperature of animals was measured every 30–60 minutes using a rectal probe for at least 2 hours, and maintained between 37–37.8°C. To avoid hypothermia, rats were placed into a heating chamber (Thermacage, Datesand Ltd, UK) maintained at 28–30°C, and subsequently housed in a holding room maintained at 26–28°C, where if necessary additional heating or cooling was performed by applying fan heating or a cold water spray.

### Animals used and sample size

Forty-six rats underwent surgery for permanent MCAO. Based on predetermined exclusion criteria, seventeen animals were excluded from the study: three animal were excluded due to an insufficient decrease in cerebral blood flow following insertion of the monofilament, three animals were excluded due to a lack of infarct lesion at 24 hours after MCAO (one treated with vehicle and two with R18), ten were excluded due to an infarct volume of ≤ 75 mm^3^ (four treated with vehicle, four with R18 and two with R18D) and one animal (R18) was excluded due to the development of a subarachnoid hemorrhage following MCAO due to filament insertion. Animals displaying an infarct volume ≤ 75 mm^3^ (or no infarct) were excluded from the study, as an infarct of this volume is considered small following permanent MCAO, and reflective of excellent cerebral collateral circulation and/or partial MCAO. For infarct volume analysis vehicle and peptide treatment groups each consisted of 9–10 animals, and includes one R18 treated animal that died several hours before the 24-hour post-MCAO study end-point. In addition, one R18 treated animal was inadvertently omitted for neurological assessment at 24-hours post-MCAO.

### Infarct volume and cerebral hemisphere swelling measurements

Infarct volume was assessed 24 hours after MCAO as previously described [47]. Briefly, 2 mm thick cerebral coronal brain slices were stained with 2,3,5 triphenyltetrazolium chloride (TTC; Sigma-Aldrich, USA) and digital images analysed using ImageJ software (3^rd^ edition, NIH, USA) by an operator blind to treatment status. The total infarct volume was determined by measuring the areas of infarcted tissue on both sides of each 2 mm slices. Infarct volume measurements were corrected for cerebral oedema using the ratio of stroke-affected hemisphere volume to contralateral hemisphere volume. The cerebral oedema ratio was also used to determine the percentage of cerebral swelling of the stroke-affected hemisphere.

### Functional assessment

In order to determine if peptide treatment improved sensorimotor outcomes, three functional tests were undertaken, as previously described [5], namely neurological assessment using the modified Bederson scale, the adhesive tape removal test and the rota-rod test. An operator blind to treatment status performed the functional tests 24 hours after MCAO.

### Tissue histology

At experiment end-point, samples of liver, kidney, lung, heart and spleen were collected from representative animals treated with vehicle, R18 and R18D and placed in 4% formalin. Tissue samples were subsequently paraffin embedded, sectioned at a thickness of 10 μm and stained with hematoxylin and eosin (H & E) before being examined by light microscopy.

### Cell cultivation

#### Cortical neuronal cultures

Establishment of rat primary cortical cultures in Neurobasal/2% B27 supplement (NB/B27; Life Technologies, Australia) using cortical tissue obtained directly from E18 day embryos was as previously described [1]. Neurons were seeded into 96-well plastic plates (Nunc, Australia), and maintained in a CO_2_ incubator (5% CO_2_, 95% air balance, 98% humidity) at 37°C until use on day *in vitro* 10 to 12. Under these conditions cultures routinely consist of >97% neurons and 1–3% astrocytes, and are highly sensitive to glutamic acid excitotoxicity.

#### Astrocyte cultures

The astrocytes were isolated from Sprague-Dawley rat pups (P2), and dissociated in Dulbeco’s Modified Eagle Medium (DMEM; Life Technologies) supplemented 1.3 mM L-cysteine, 0.9 mM NaHCO_3_, 10 units/mL papain (Sigma-Aldrich), 50 μ/mL Dnase (Sigma-Aldrich), and washed with DMEM/10% horse serum. The dissociated cells were resuspended in DMEM/10% foetal bovine serum (FBS) and transferred to a 25cm^2^ culture flask for maintenance in a CO_2_ incubator at 37°C until reaching confluency after 12–14 days. Following the trypsinisation of astrocytes in flasks, cells were seeded into 96-well culture plates (25,000 cells per well) and used 2–3 days later for toxicity studies.

#### Human brain endothelial (b.END3) and human embryonic kidney 293 cells (HEK293)

b.End.3 and HEK293 cell cultures were maintained in 25cm^2^ culture flasks in DMEM/5% FBS in a CO_2_ incubator at 37°C. Following the trypsinisation of cell monolayers in flasks, cells were seeded at a density of 20,000 to 30,000 cells per well (96-well plate) and used 1–2 days later for toxicity studies.

### *In vitro* assessment of R18 and R18D peptide toxicity

Cultured cells were exposed to R18 and R18D (0.4 to 25.6 μM) by replacing media in wells with a 100 μL volume of NB/B27 (neurons) or DMEM/5% FBS (astrocytes, bEnd.3 and HEK293) containing the appropriate concentration of peptide. Cells were incubated in a CO_2_ incubator at 37°C for either 10 minutes or 24 hours. After the 10 minutes peptide incubation, culture media was replaced with either NB/B27 or DMEM/5% FBS and incubated in a CO_2_ incubator at 37°C for a further 24 hours. Peptide toxicity was assessed approximately 24 hours after the initial peptide exposure by measuring cell viability by MTS (3-(4,5,dimethyliazol-2-yl)-5-(3-carboxymethoxy-phenyl)-2-(4-sulfophenyl)-2H-tetrazolium salt) assay (Promega, Australia) and LDH (lactate dehydrogenase) release assay (ThermoFisher Scientific, Australia). For MTS and LDH assay’s absorbance values for untreated control cultures were considered to represent 100% cell viability, while wells treated with sodium dodecyl sulphate (SDS; 10% w/v; 10μl per 100μl) were considered to represent 100% cell death. A significant (*p <* 0.05) decrease or increase in MTS and/or LDH assay absorbance values respectively, from untreated control cultures following R18 or R18D treatment was considered a toxic effect.

### Statistical analysis

Data for physiological parameters, infarct volume, cerebral hemisphere swelling, adhesive tape removal, rota-rod and cell viability were evaluated by ANOVA followed by Fisher’s post-hoc analysis. Data from the neurological assessment measurements were analysed using the Mann-Whitney test. A value of *p <* 0.05 was considered statistically significant. MTS and LDH absorbance (490 nm) data was analysed by ANOVA, followed by post-hoc Fischer’s PLSD test, with *p <* 0.05 values considered statistically significant. At least four wells were used in all assays and usually repeated a minimum of two times independently.

## Results

### Physiological data, infarct volume, cerebral hemisphere swelling, tissue histology

**Fig 1:**
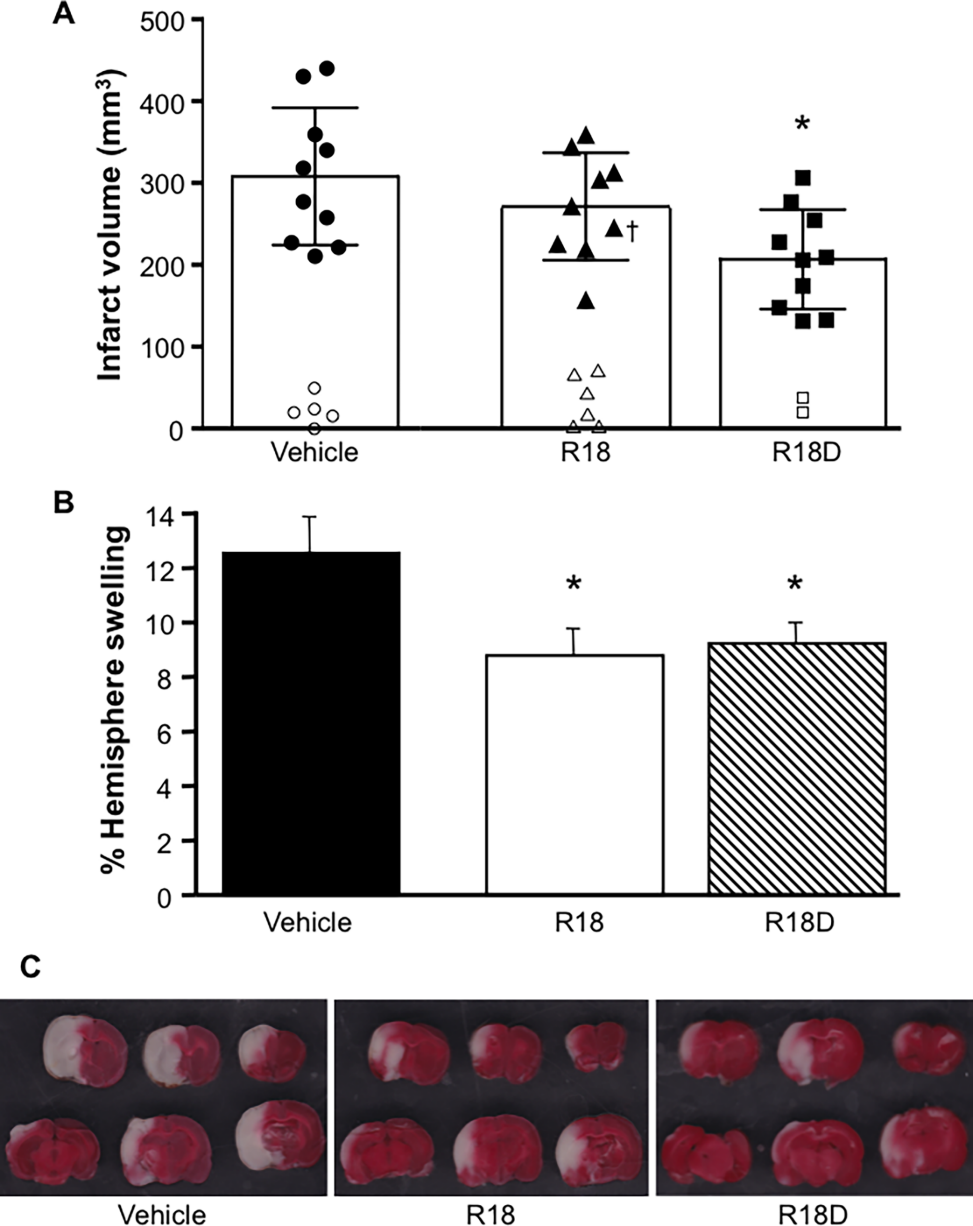
Infarct volume analysis, cerebral hemisphere swelling and representative images of coronal brain slices as assessed 24-hours after permanent MCAO. Treatments were administered intravenously (saline vehicle, or R18 and R18D at 300 nmol/kg) 30 min after MCAO. A: Infarct volume measurements. ^†^Denotes animal that died several hours before the 24-hour post-MCAO study end-point, but was still included in the final infarct volume analysis. Unfilled circles, triangles and squares represent infarct volumes obtained from outlier animals excluded from the study. B: Percentage hemisphere swelling for vehicle and the R18 and R18D treatment groups. C: Representative images of TTC stained coronal brain slices from vehicle, R18 and R18D treatment groups. Values are mean ± SD; n = 9–10. *p < 0.05 when compared to vehicle.

Animal physiological parameters measured before and during MCAO did not show significant differences between treatment groups (Table 1). Data for infarct volume measurements and percentage cerebral hemisphere swelling are presented in Fig 1(A) and 1(B), and representative TTC stained coronal brain slices in Fig 1(C). When compared with the vehicle, R18D treatment reduced infarct volume by 33% (*p =* 0.004), while R18 treatment reduced infarct volume by 12% (*p =* 0.27). Both R18 and R18D significantly reduced hemisphere swelling by 30% (*p =* 0.02) and 27% (*p =* 0.03), respectively. Light microscopic examination of H & E stained sections of kidney, liver, spleen, and heart obtained from saline, R18 or R18D treated animals did not reveal any obvious abnormal histological features (see S1 Fig for liver and kidney sections). While lung tissue appeared normal, in some areas there was evidence of a cellular infiltrate within the alveolar space, but this was present in saline, R18 and R18D treated animals and may be related to the anaesthesia and ventilation of the animal during surgery.

**Table 1:** Physiological parameters.

Parameter	Experimental groups		
	Saline	R18	R18D
PaO_2_ (mmHg)	126 ± 13.22	116 ± 23.19	117 ± 34.15
PaCO_2_ (mmHg)	42 ± 4.25	39 ± 9.36	43 ± 3.77
pH	7.35 ± 0.07	7.36 ± 0.09	7.4 ± 0.03
Glucose (mmol/L)	5.3 ± 0.37	4.8 ± 1.86	6.7 ± 1.73
BP (mmHg; average during surgery)	87.15 ± 9.48	96 ± 3.49	90 ± 8.12
Temperature (°C; average during surgery)	37.29 ± 0.36	37.17 ± 0.31	37.3 ± 0.24
Temperature (°C; average 2h post-surgery)	37.61 ± 0.28	37.56 ± 0.25	37.63 ± 0.19

PaO_2_, PaCO_2,_ pH and glucose measured before MCAO. All values are expressed as mean ± SD.

### Functional outcomes and weight loss measurements

**Fig 2:**
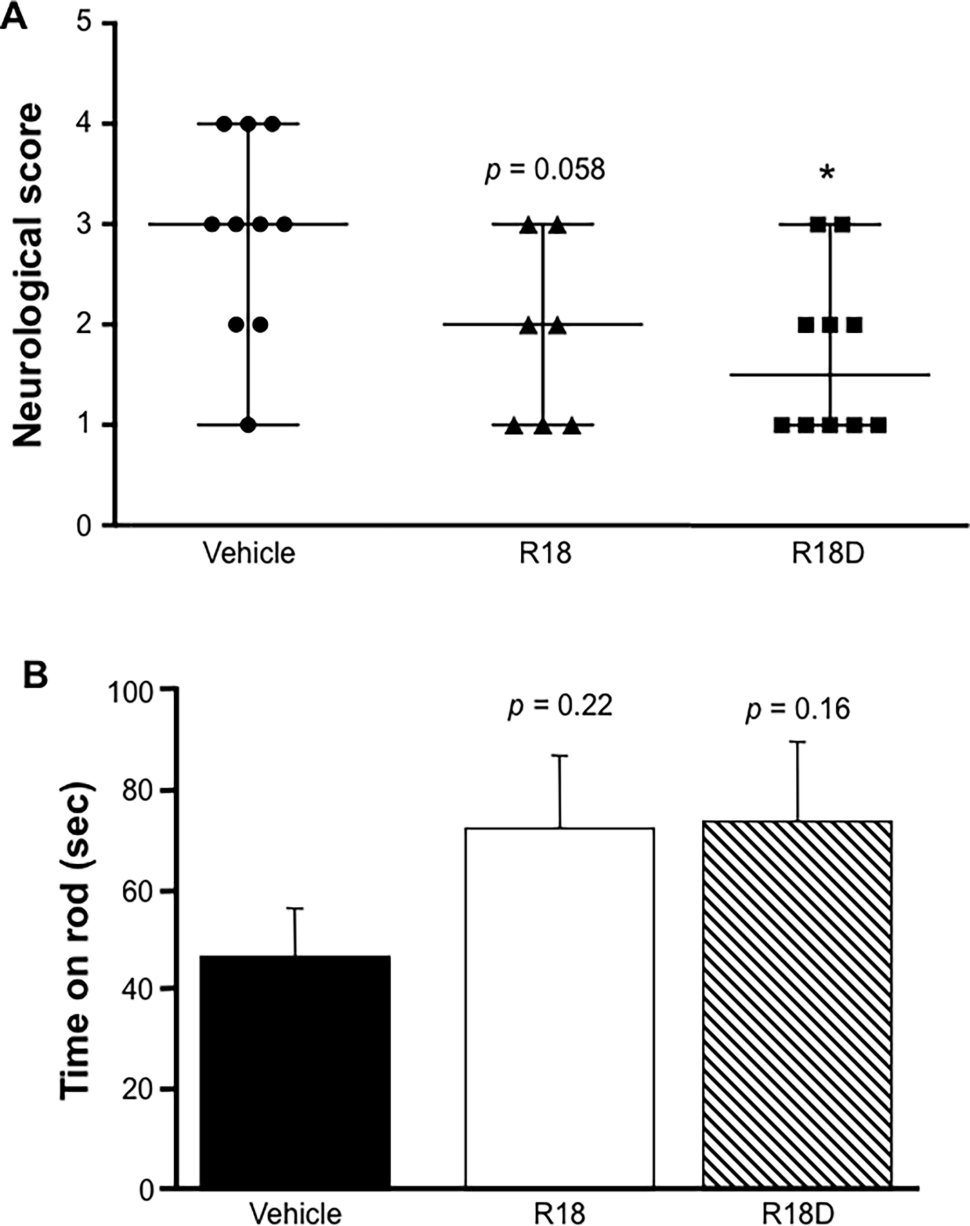
Neurological score and rota-rod measurements, 24-hours after permanent MCAO. A. Neurological grading scores (0 = no deficit, 5 = major deficit) for vehicle (saline), R18 and R18D (300 nmol/kg) treatment groups. Vertical lines on graph indicate the range and median for neurological score; n = 7–10. *p < 0.05, when compared to the vehicle. Note: one R18 treated animal inadvertently did not undergo neurological assessment. B. Rota-rod performance for vehicle, R18 and R18D treatment groups. Sec = seconds. Values are mean ± SD; n = 8–10.

**Fig 3:**
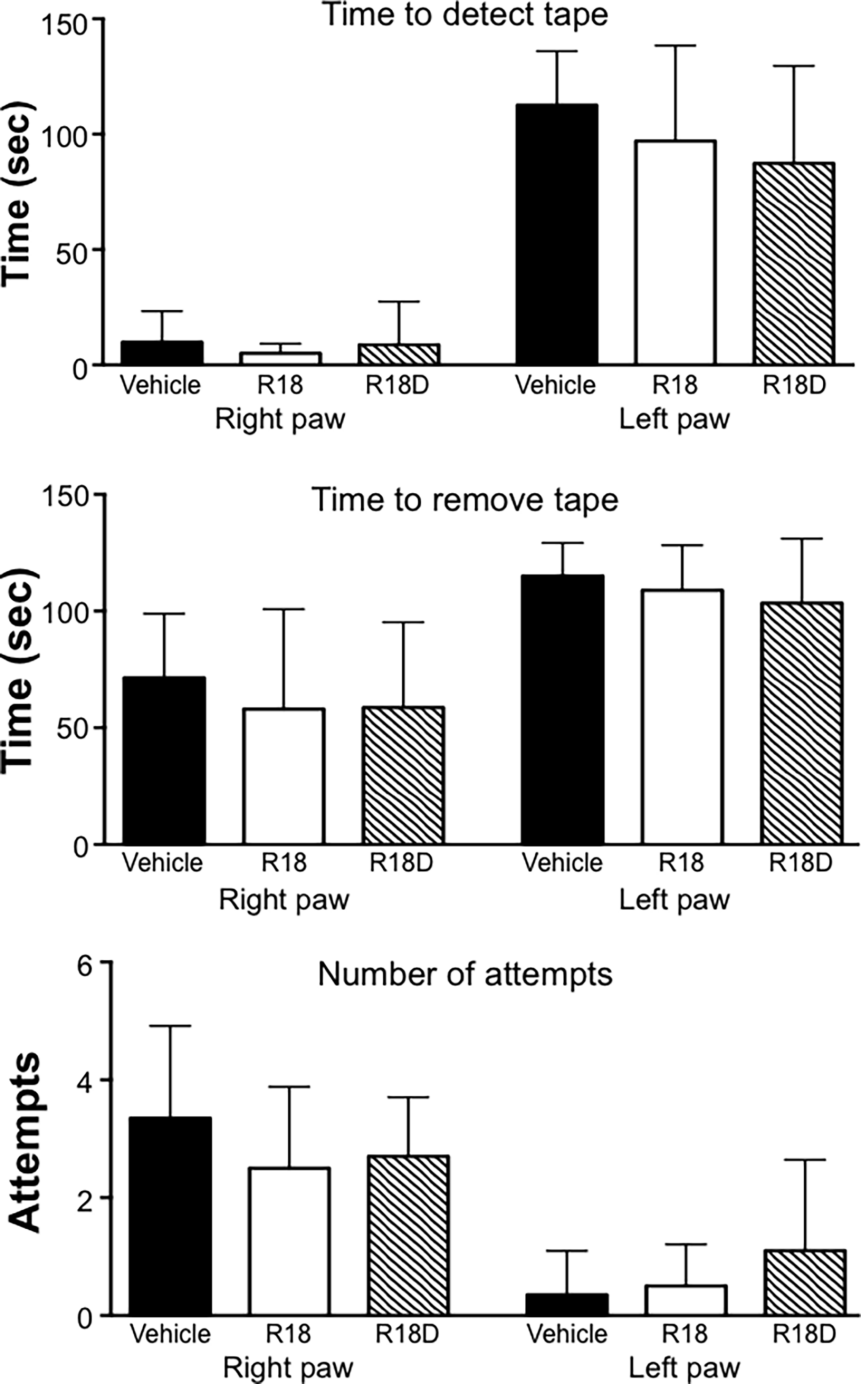
Adhesive tape test measurements 24-hours after permanent MCAO. Treatment groups are vehicle (saline), R18 and R18D (300 nmol/kg). Time (sec = seconds) to detect tape, time to remove tape and number of attempts to remove tape. Values are mean ± SD; n = 8–10. Maximum time allowed for adhesive tape removal was 120 seconds.

When compared to the vehicle, the R18D treatment group showed a significantly improved neurological score (*p =* 0.02), while the R18 treatment group showed a trend to improvement in neurological score (*p =* 0.058; Fig 2A). Although not statistically significant, R18 and R18D treatment groups displayed a trend towards improvements in rota-rod performance (Fig 2B). Measurements for the adhesive tape test did not reveal any significant improvements, although the R18D treatment group displayed positive trends for the time to detect tape, time to remove tape and number of attempts to remove tape (Fig 3). As expected, the left paw was more adversely affected than the right paw. All treatment groups recorded a loss in body weight 24-hours post-MCAO from 34g (11.3%) for vehicle, 32g (10.6%) for R18 and 28.7g (9.6%) for R18D (S2 Fig).

### Assessment of R18 and R18D toxicity in cell culture

#### Cortical neurons

**Fig 4:**
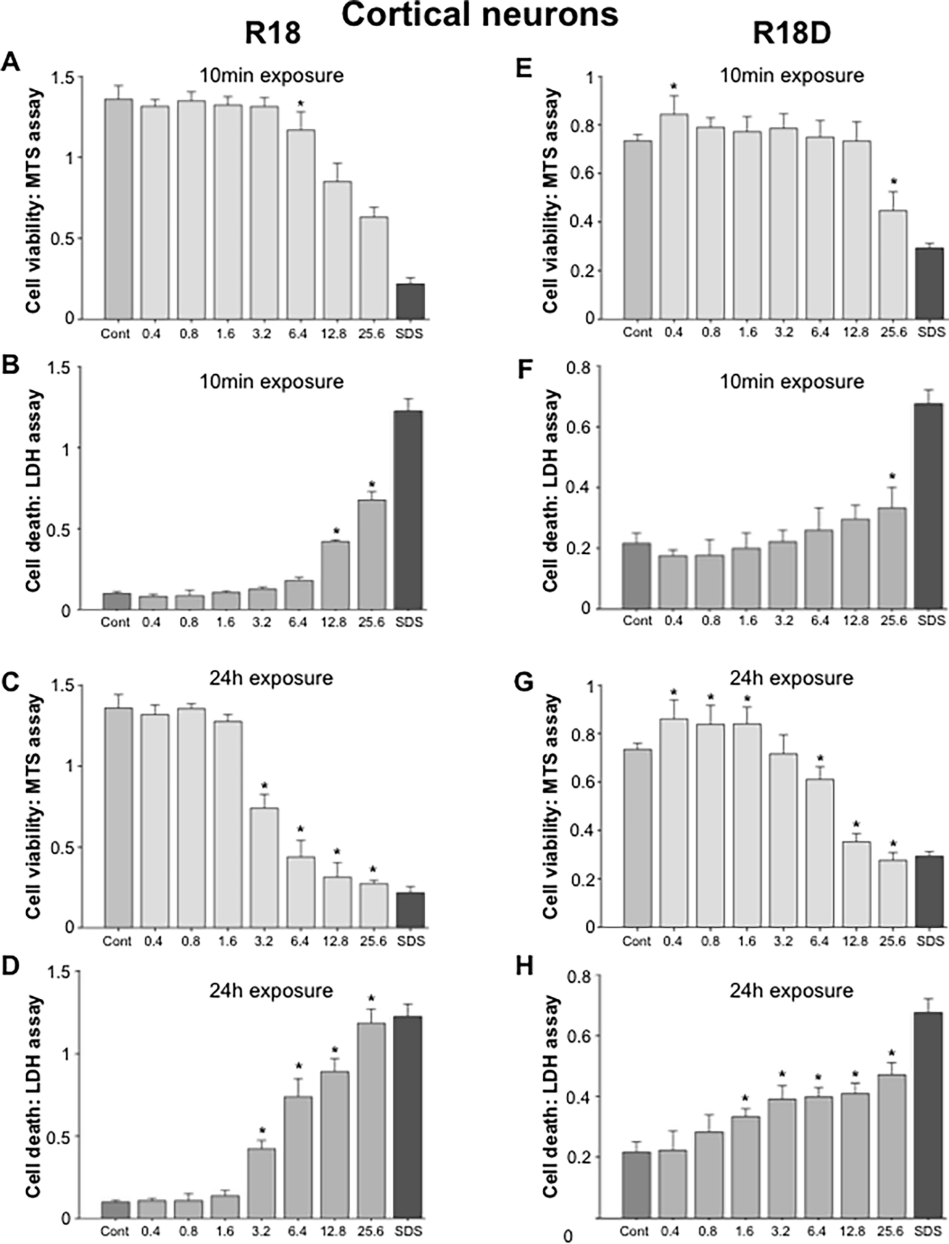
Cell viability and cell death in cortical neuronal cell cultures following exposure to different concentrations of R18 or R18D peptide for either 10 minutes or 24 hours as measured by MTS and LDH assays. Peptide concentration in μM. Cell viability was measured 24-hours after the addition of peptide. A, B, C, D: R18 and E, F, G, H: R18D. *p < 0.05 when compared to control [Cont = 100% cell viability (MTS assay) or 0% cell death (LDH assay)]. SDS = wells treated with SDS [SDS = 0% cell viability (MTS assay) or 100% cell death (LDH assay)]. Values are means ± SE; n = 4.

Following a 10-minute exposure of neuronal cultures to R18 a significant toxic effect was detected starting at 6.4 μM (Fig 4A and 4B), while for R18D a toxic effect was detected at 25.6 μM only (Fig 4E and 4F). Incubation with either R18 or R18D for a 24-hour duration resulted in toxicity starting at 3.2 μM and 1.6 μM, respectively (Fig 4C, 4D, 4G and 4H). Interestingly R18D at some concentrations, especially following a 24-hour exposure increased MTS values above untreated controls (Fig 4E and 4G).

#### Astrocytes

**Fig 5:**
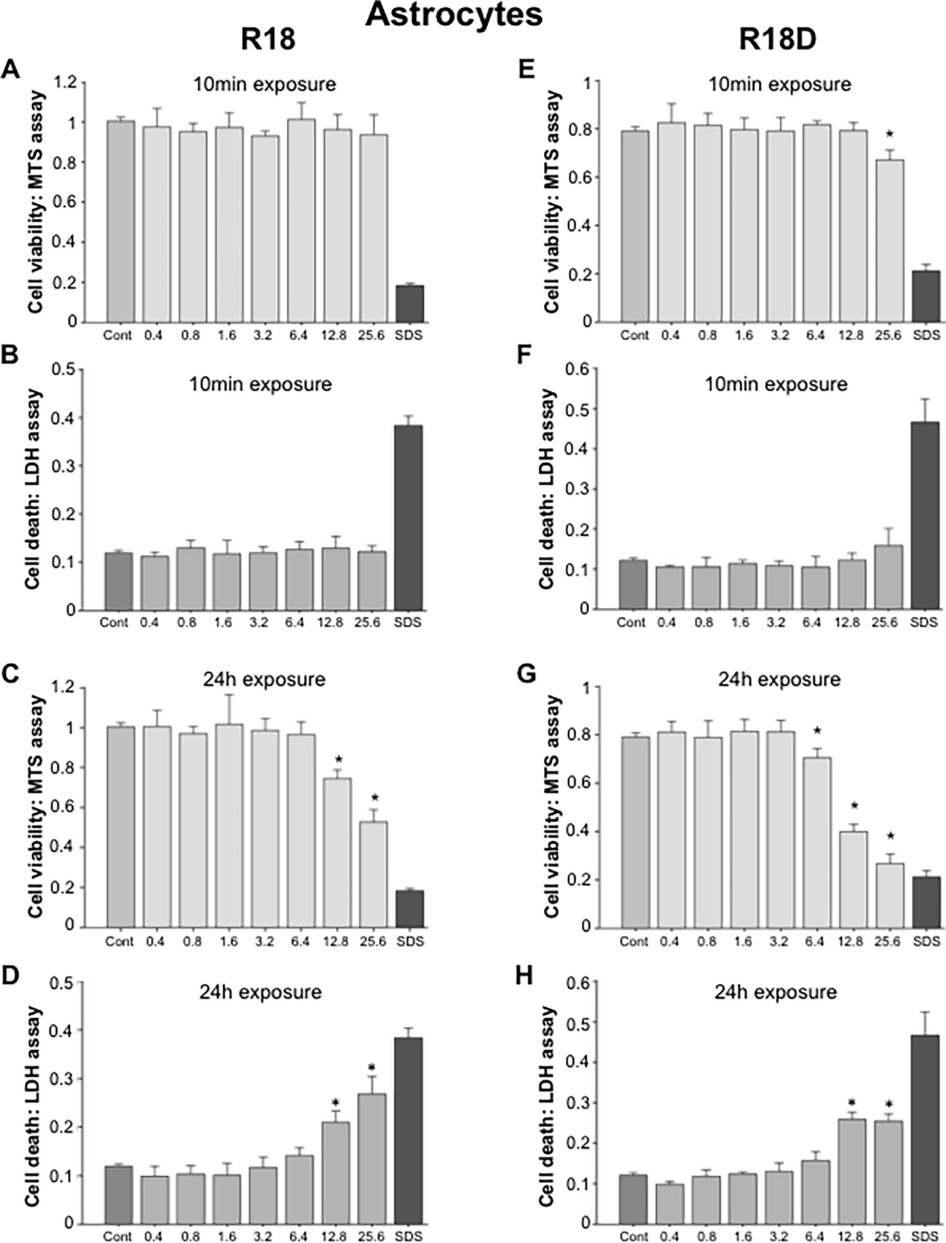
Cell viability and cell death in astrocyte cell cultures following exposure to different concentrations of R18 or R18D peptide for either 10 minutes or 24 hours as measured by MTS and LDH assays. Peptide concentration in μM. Cell viability was measured 24-hours after the addition of peptide. A, B, C, D: R18 and E, F, G, H: R18D. *p < 0.05 when compared to control [Cont = 100% cell viability (MTS assay) or 0% cell death (LDH assay)]. SDS = wells treated with SDS [SDS = 0% cell viability (MTS assay) or 100% cell death (LDH assay)]. Values are means ± SE; n = 4.

A 10-minute exposure of astrocyte cell cultures to R18 did not cause any detectable toxicity at any of the concentrations examined (Fig 5A and 5B), while a significant toxic effect for R18D was only detected at 25.6 μM (Fig 5E and 5F). In comparison, a 24-hour exposure of astrocyte cell cultures to R18 or R18D resulted in toxicity starting at 12.8 μM and 6.4 μM, respectively (Fig 5C, 5D, 5G and 5H).

#### bEnd.3 cells

**Fig 6:**
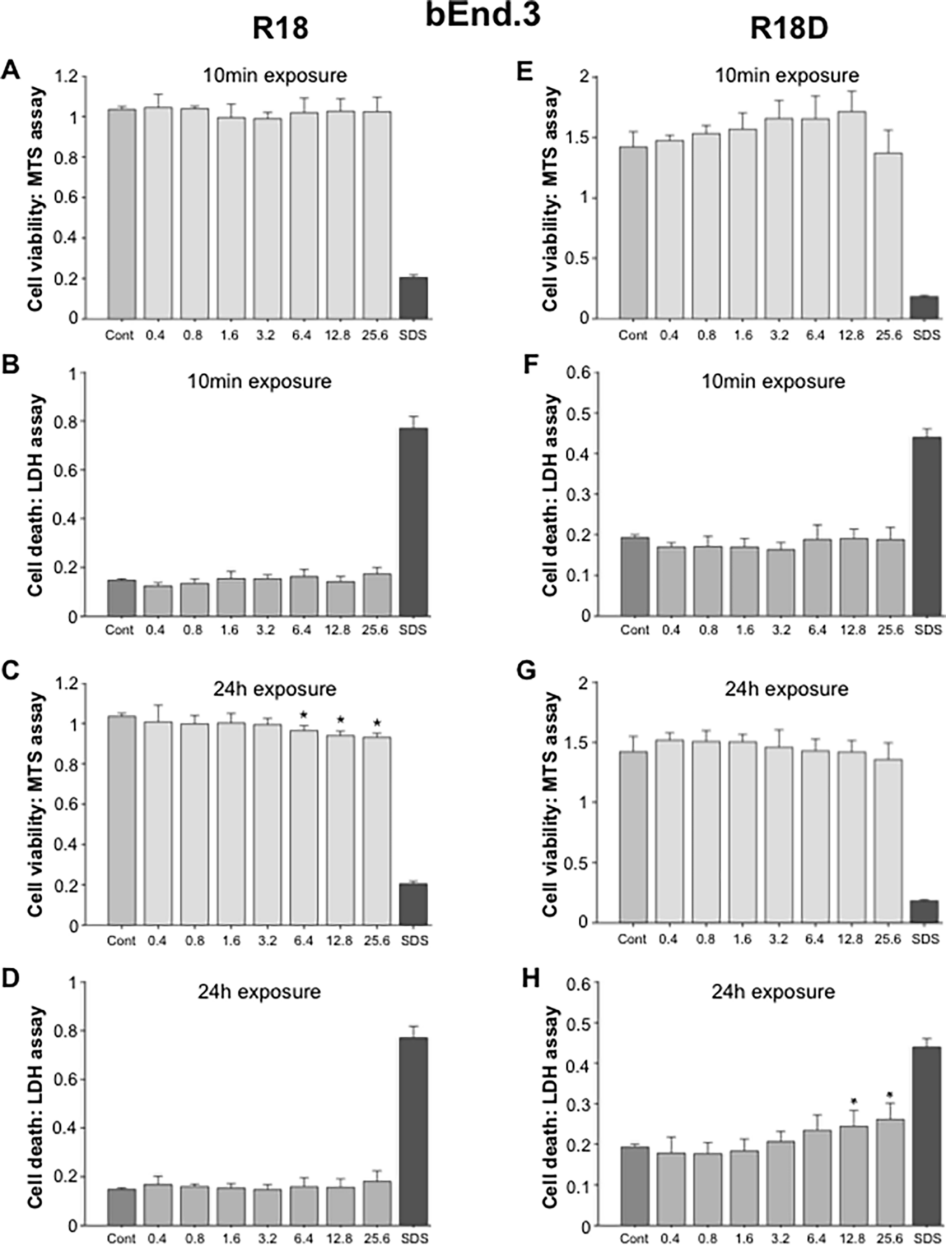
Cell viability and cell death in b.End3 brain endothelial cell cultures following exposure to different concentrations of R18 or R18D peptide for either 10 minutes or 24 hours as measured by MTS and LDH assays. Peptide concentration in μM. Cell viability was measured 24-hours after the addition of peptide. A, B, C, D: R18 and E, F, G, H: R18D. *p < 0.05 when compared to control [Cont = 100% cell viability (MTS assay) or 0% cell death (LDH assay)]. SDS = wells treated with SDS [SDS = 0% cell viability (MTS assay) or 100% cell death (LDH assay)]. Values are means ± SE; n = 4.

A 10-minute exposure of bEnd.3 cell cultures to R18 or R18D did not cause any detectable toxicity at any of the concentrations examined (Fig 6A, 6B, 6E and 6F). In comparison, a 24-hour exposure of bEnd.3 cell cultures to R18 or R18D resulted in toxicity starting at 6.4 μM and 12.8 μM, respectively (Fig 6C, 6D, 6G and 6H).

#### HEK293 cells

**Fig 7:**
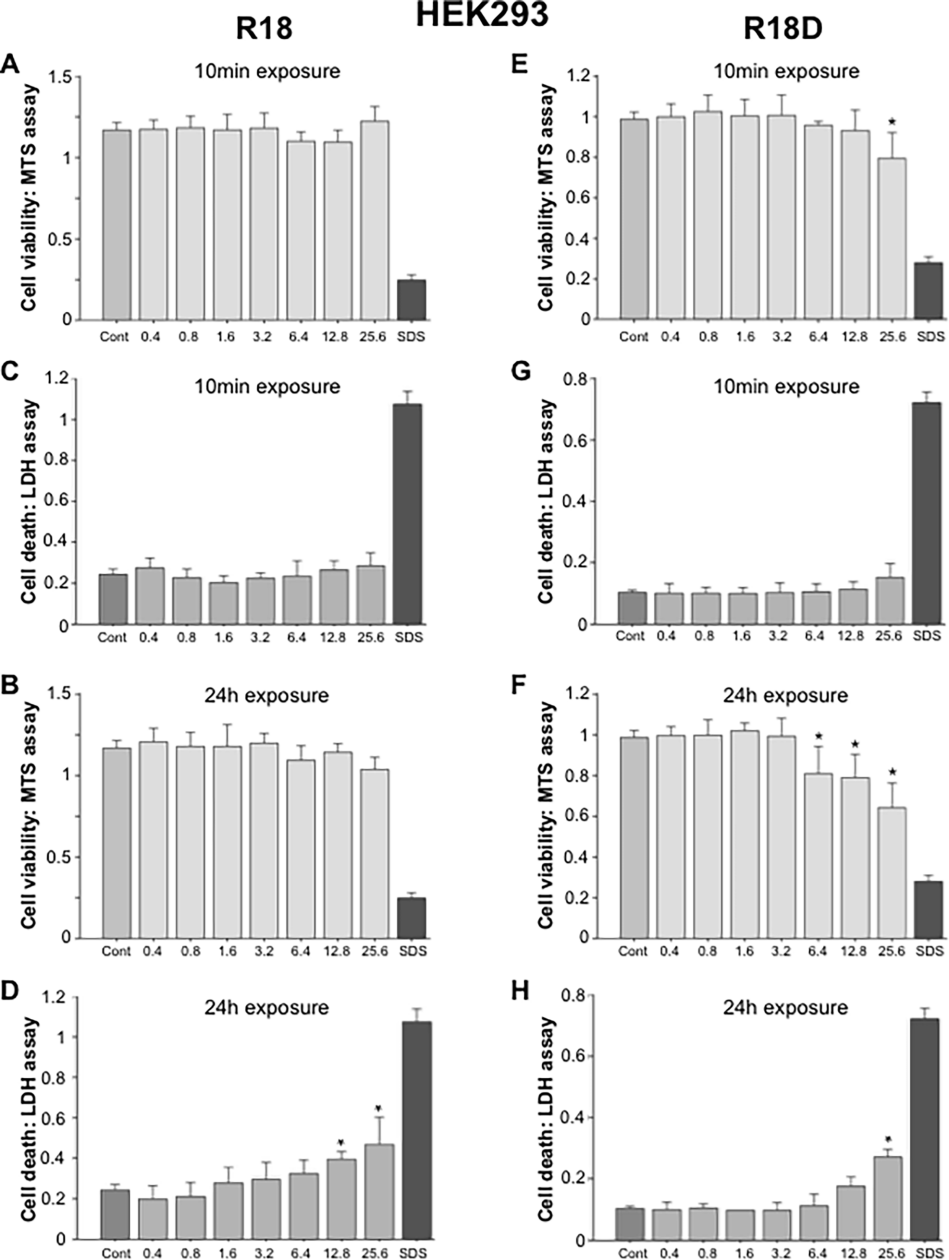
Cell viability and cell death in HEK293 embryonic kidney cell cultures following exposure to different concentrations of R18 or R18D peptide for either 10 minutes or 24 hours as measured by MTS and LDH assays. Peptide concentration in μM. Cell viability was measured 24-hours after the addition of peptide. A, B, C D: R18 and E, F, G, H: R18D. *p < 0.05 when compared to control [Cont = 100% cell viability (MTS assay) or 0% cell death (LDH assay)]. SDS = wells treated with SDS [SDS = 0% cell viability (MTS assay) or 100% cell death (LDH assay)]. Values are means ± SE; n = 4.

A 10-minute exposure of HEK293 cell cultures to R18 did not cause any detectable toxicity at any of the concentrations examined (Fig 7A and 7B), while a significant toxic effect for R18D was only detected at 25.6 μM (Fig 7E and 7F). In comparison, a 24-hour exposure of HEK293 cell cultures to R18 or R18D resulted in toxicity starting at 12.8 μM and 6.4 μM, respectively (Fig 7C, 7D, 7G and 7H).

## Discussion

The primary objective of the present study was to compare the neuroprotective efficacy of L- and D-isoforms of the R18D peptide in a permanent MCAO stroke model in the Wistar rat. Using a peptide dose of 300 nmol/kg administered 30 minutes after MCAO, R18D demonstrated a greater capacity to reduce infarct volume compared to R18, while both peptides reduce cerebral hemisphere swelling and improve functional outcomes to a similar degree. These findings are in line with our previous studies demonstrating the effectiveness of poly-arginine peptides in animal stroke models [2,4–7], and highlight the potential superior neuroprotective potency of the more stable D-enantiomer of R18. However the 24-hour end-point and the use of a single dose of the peptides are limitations of the study, and therefore additional R18 and R18D dose response studies in both permanent and transient MCAO stroke models examining long-term histological and functional end-points are required to confirm the findings. The study has for the first time also confirmed the neuroprotective efficacy of a poly-arginine peptide in a strain of rat (Wistar) other than Sprague Dawley.

In addition to the confirmed neuroprotective properties of CARPs [2–4, 8–12, 25–39], they possess other features that are potentially beneficial for neuroprotection. CARPs have been demonstrated to down-regulate cell death TNF receptor proteins [48], target and stabilise mitochondria [49–52], inhibit the activity of the proteasome [53–55], reduce lipid peroxidation [56] and scavenge free radicals [57], modulate immune and inflammatory responses [56,58–62], and inhibit proprotein convertases that activate matrix metalloproteinases [63–65]. For example, matrix metalloproteinase inhibition may explain the capacity of CARPs to reduce the hemisphere swelling observed in the present and our previous studies [4,7] due to blood brain barrier integrity maintenance.

The superior efficacy of the D-enantiomer R18 peptide is significant as the increased potency of the peptide could also enhance its neuroprotective effectiveness at the clinical level. While the exact reasons for the increased potency of R18D versus R18 are not fully known, it is likely that due to its increased resistance to enzymatic degradation, R18D has better pharmacokinetic properties in terms of stability in serum, as well as in extracellular and intracellular compartments. Conversely, increased peptide stability could increase the risk for inducing cellular toxicity, especially at high peptide doses in already stressed cells. In this regard, it is encouraging that the D-enantiomer R9 peptide (ALX40-4C) did not cause any serious side effects when administered intravenously at 480 nmol/kg 3 days per week for 4 weeks in asymptomatic HIV patients [66].

With respect to peptide toxicity, in the present study histological assessment of various tissues collected from animals 24 hours after peptide administration did not disclose any treatment specific abnormalities. Peptide toxicity results based from cell culture studies were highly dose, time and cell-type dependent. Neurons were the most sensitive to the toxic effects of the peptides, followed by astrocytes, HEK293 cells and bEND.3 cells. Comparatively, the toxic effects induced by R18 and R18D were similar across the different peptide concentrations, exposure durations and cell types. Furthermore it is unlikely that the lowest peptide concentration causing neuronal toxicity *in vitro* (R18D: 1.6 μM/24h), would be achieved in the extracellular space within the brain following an intravenous dose of 300 nmol/kg (equals 0.3 μM if peptide evenly distributed), due to low uptake into the brain and greater potential for peptide clearance *in vivo*, but this would need to be confirmed. Interestingly, as we have previously observed in *in vitro* studies with other poly-arginine peptides, based on MTS assay measurements low concentrations of R18D, appeared to increase neuronal viability, especially following a 24-hour duration of exposure. The increased neuronal viability could reflect reduced background cell death and/or an improved ability of neurons to reduce MTS, however based on both MTS and LDH data it appears the peptide is improving neuronal metabolism. Since CARP’s can target mitochondria it is possible R18D is improving mitochondrial bio-energetics, and thereby increasing the ability of neurons to reduce MTS.

In conclusion, we have demonstrated that at the 300 nmol/kg dose, R18D is more effective than its L-isomer R18 in reducing infarct volume after MCAO in the Wistar rat stroke model, whereas both peptides reduced cerebral hemisphere swelling and had a positive effect on functional outcomes. Moreover there was no indication of *in vivo* toxicity at the therapeutic dose used, while *in vitro* studies showed evidence of cellular toxicity only with high peptide concentrations and prolonged exposure times. The findings have clinical significance and justify further investigation of the R18 peptide as a novel class of neuroprotective agent for stroke and other forms of acute and chronic brain injury.

## Supporting information

S1 Fig Photomicrographs of histological sections of kidney and liver tissue. Representative H & E stained 10 μm sections obtained from vehicle (saline), R18 or R18D (300 nmol/kg) treated animals approximately 24 hours after treatment administration. Magnification X400.
(PPT)

S2 Fig Weight loss at 24-hours after permanent MCAO. Treatment groups are vehicle (saline), R18 and R18D (300 nmol/kg). Values are mean ± SD; n = 8–10. Note: an animal that died several hours before the 24-hour post-MCAO study end-point was not included.
(PPT)
