# Supplementary figures and images for: Comparison of neuroprotective efficacy of poly-arginine R18 and R18D (D-enantiomer) peptides following permanent middle cerebral artery occlusion in the Wistar rat and in vitro toxicity studies
Source: PLoS One. 2018 Mar 7;13(3):e0193884. doi: 10.1371/journal.pone.0193884 (PMC5841795; doi:10.1371/journal.pone.0193884)

## Slide 1
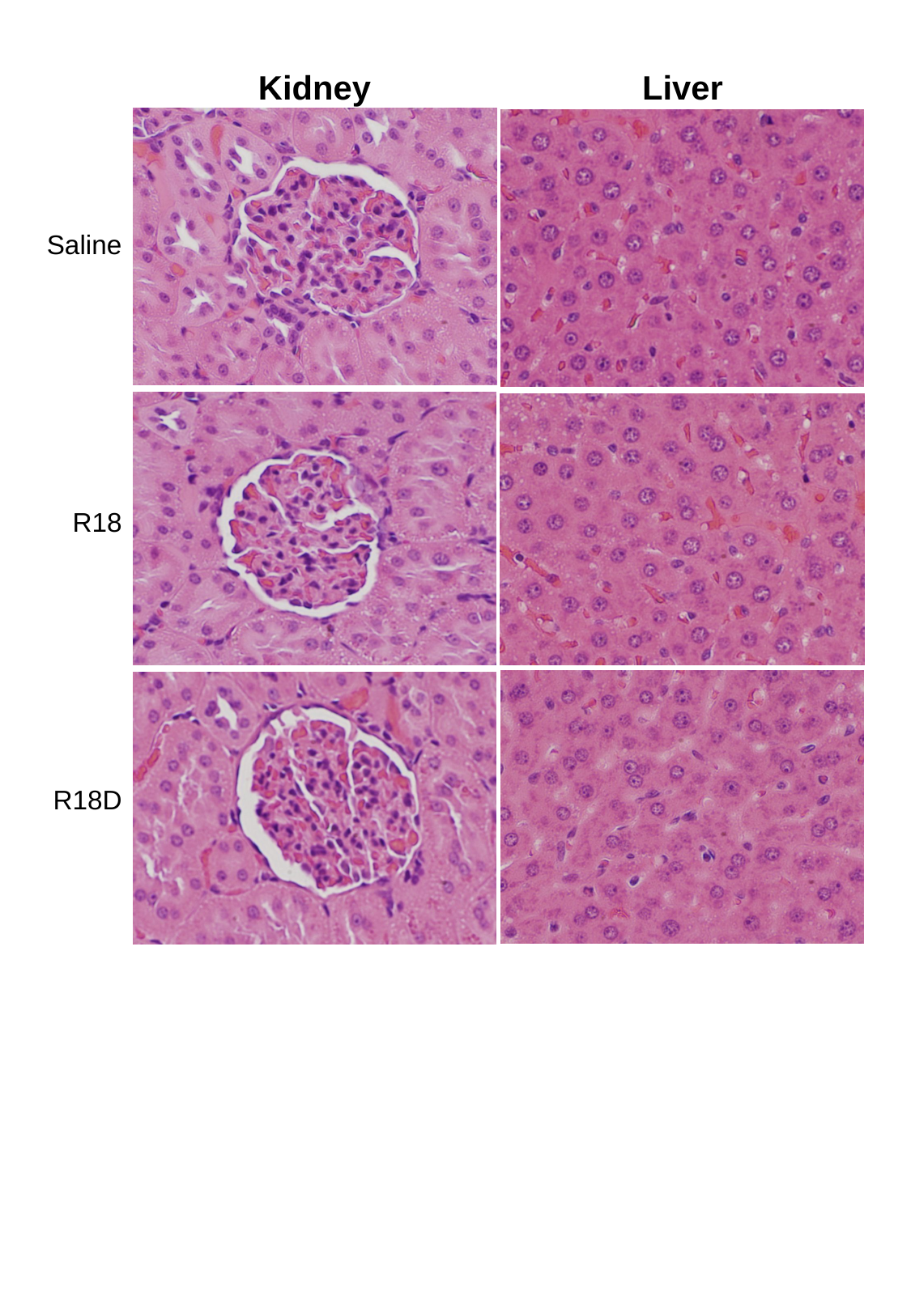

Kidney
Liver
Saline
R18
R18D

Supplement: S1 Fig — Representative H & E stained 10 μm sections obtained from vehicle (saline), R18 or R18D (300 nmol/kg) treated animals approximately 24 hours after treatment administration. Magnification X400. (PPT) [file pone.0193884.s001.ppt]

## Slide 1
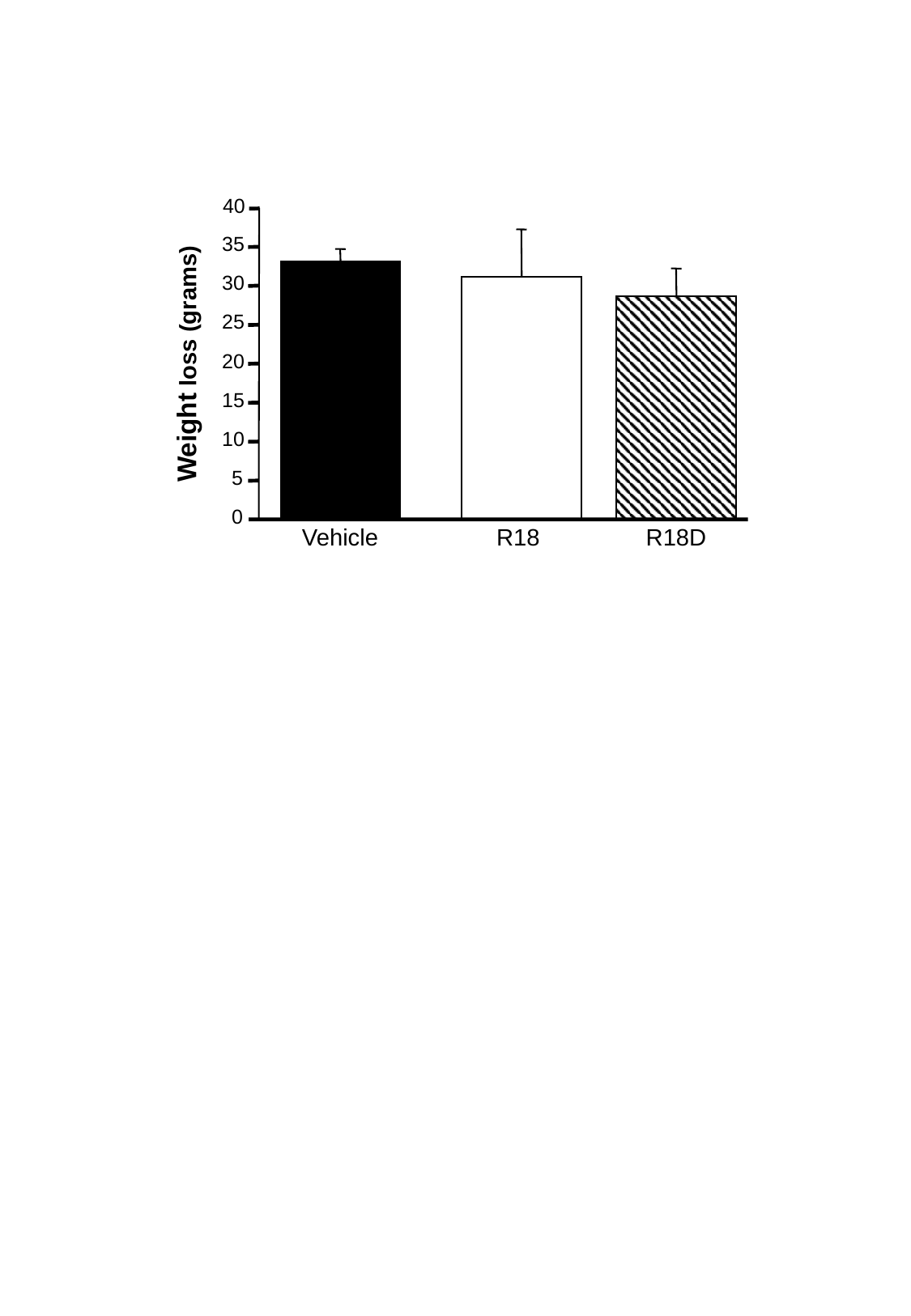

40
35
30
25
Weight loss (grams)
20
15
10
5
0
Vehicle
R18
R18D

Supplement: S2 Fig — Treatment groups are vehicle (saline), R18 and R18D (300 nmol/kg). Values are mean ± SD; n = 8–10. Note: an animal that died several hours before the 24-hour post-MCAO study end-point was not included. (PPT) [file pone.0193884.s002.ppt]
